# Genetic Diversity of Serine Protease Inhibitors in Myxozoan (Cnidaria, Myxozoa) Fish Parasites

**DOI:** 10.3390/microorganisms8101502

**Published:** 2020-09-29

**Authors:** Edit Eszterbauer, Dóra Sipos, Győző L. Kaján, Dóra Szegő, Ivan Fiala, Astrid S. Holzer, Pavla Bartošová-Sojková

**Affiliations:** 1 Institute for Veterinary Medical Research, Centre for Agricultural Research, 1143 Budapest, Hungary; sipos.dora@atk.hu (D.S.); kajan.gyozo@atk.hu (G.L.K.); szego.dora@atk.hu (D.S.); 2 Institute of Parasitology, Biology Centre of the Czech Academy of Sciences, 370 05 České Budějovice, Czech Republic; fiala@paru.cas.cz (I.F.); astrid.holzer@paru.cas.cz (A.S.H.); bartosova@paru.cas.cz (P.B.-S.)

**Keywords:** serpins, microscopic parasite, free-living Cnidaria, phylogeny, signature, conserved domains, therapeutic target

## Abstract

We studied the genetic variability of serine protease inhibitors (serpins) of Myxozoa, microscopic endoparasites of fish. Myxozoans affect the health of both farmed and wild fish populations, causing diseases and mortalities. Despite their global impact, no effective protection exists against these parasites. Serpins were reported as important factors for host invasion and immune evasion, and as promising targets for the development of antiparasitic therapies. For the first time, we identified and aligned serpin sequences from high throughput sequencing datasets of ten myxozoan species, and analyzed 146 serpins from this parasite group together with those of other taxa phylogenetically, to explore their relationship and origins. High intra- and interspecific variability was detected among the examined serpins. The average sequence identity was 25–30% only. The conserved domains (i.e., motif and signature) showed taxon-level differences. Serpins clustered according to taxonomy rather than to serpin types, and myxozoan serpins seemed to be highly divergent from that of other taxa. None of them clustered with their closest relative free-living cnidarians. The genetic distinction of myxozoan serpins further strengthens the idea of an independent origin of Myxozoa, and may indicate novel protein functions potentially related to parasitism in this animal group.

## 1. Introduction

Myxozoans are microscopic parasites with simplified body organization. They are close relatives of free-living Cnidaria such as jellyfish, sea anemones, and hydras, but diverged from the common ancestor in ancient times to become endoparasites that today comprise the Myxozoa [1]. During a complex two-host life cycle, they infect both vertebrates (mostly fish) and invertebrates (mostly annelid worms) in freshwater and marine environments worldwide. The biodiversity of these parasites is remarkable and a result of host-associated cospeciation [2,3,4]. Developmental stages of myxozoans are motile during invasion, migration, and proliferation due to cellular processes that enable unique cell motility [5,6,7,8]. Myxozoans affect the health of both farmed and wild fish populations, causing diseases and mortality. Despite their global impact, effective protection of fish is not available against these parasites [1].

Serine protease inhibitors (serpins, classified as inhibitor family I4) are the largest and most broadly distributed superfamily of protease inhibitors [9,10]. Serpin-like genes have been detected in animals, poxviruses, plants, bacteria, and over 1500 members of this family have been identified to date. Analysis of the available genomic data revealed that most multicellular eukaryotes have serpins, including blood-feeding arthropods or venomous animals [9,10,11,12,13,14,15]. The majority of serpins inhibit serine proteases, but serpins that inhibit caspases and papain-like cysteine proteases have also been identified [9,16]. Serpins have an unusual inhibitory mechanism of action. The active site of the target protease is disrupted by the inhibitor protein undergoing an irreversible conformational change [17,18].

Serpins play an important role in the biology of parasitic organisms [19]. Schistosome serpins identified in the blood fluke *Schistosoma mansoni* are involved in both post-translational regulation of schistosome-derived proteases, as well as in parasite defense mechanisms against the action of host proteases [20]. In nematodes, serpins interact with endogenous parasite proteinases, counteract the digestion by host proteinases, inhibit the host immune response, or even act as immunomodulators [21,22]. Proteases and their inhibitors have been reported as important factors for invasion and immune evasion of parasites [23,24,25,26]. The disparate structure of parasite serpins compared to the host orthologous proteins makes this protein family a promising target for the development of antiparasitic therapies.

Previous studies suggested that myxozoans possess serpins. On the basis of genomic and transcriptomic data, Yang et al. [27] found that 19 protease inhibitors were putatively secreted by the highly virulent myxozoan parasite, *Thelohanellus kitauei*. The majority of these (14) were serine or cysteine protease inhibitors, which may regulate the activity of such host proteases. Moreover, they revealed that the serpin family diversified extensively in the genome of *T. kitauei* compared to that of the free-living cnidarians [27]. Myxozoan genomes are extremely reduced. Recent studies reported that the genome of *Kudoa iwatai* is one of the smallest animal genomes (22.5 Mb) [28], and *Henneguya salminicola* (estimated genome size 60 Mb) even lacks mitochondrial genome. Despite the reduced genome size, serpins were detected in the genome of *H. salminicola* [29]. These findings along with their extensive diversification in some myxozoan species [27], exemplify the potential importance of serpins for parasite survival in the host.

In the present study, we identified serpins in the genomes and transcriptomes of various myxozoan species. For the first time, we examined their genetic diversity, analyzed their phylogenetic relationships within the Myxozoa, and compared them with serpins of other taxa from Protista and Animalia. Furthermore, the amino acid (aa) residues of conserved domains were investigated in detail.

## 2. Materials and Methods

### 2.1. Sequence Database Mining

All public databases of myxozoan genomes and transcriptomes were mined for serpins. The limited number of available datasets included *Myxobolus pendula* (BioProject No. PRJNA296504) [30]; *Myxobolus cerebralis* (BioProject No. PRJNA182728 [31], and PRJNA258474 [28]); *Kudoa iwatai* (BioProject Nos. PRJNA261422 and PRJNA261052) [28]; *Henneguya salminicola* (BioProject No. PRJNA485580) and *Myxobolus squamalis* (BioProject No. PRJNA485581) [29]; *Ceratonova shasta* (BioProject No. PRJNA241036) [32]; *Sphaerospora molnari* (BioProject No. PRJNA522909) [33]; *Thelohanellus kitauei* (BioProject No. PRJNA193083) [27]; *Enteromyxum leei* (BioProject No. PRJNA284325); and *Sphaeromyxa zaharoni* (BioProject No. PRJNA284326) [28]. Sequence data mining was also performed on publicly not yet available genome and transcriptome databases of *Myxobolus pseudodispar* and *Myxidium lieberkuehni*, respectively.

The local databases were screened with tblastx, and blastn/tblastx algorithms implemented in Geneious Prime 2019 using default settings (max E-value: 10), and annotated serpin sequences of *T. kitauei* as queries, to identify serpin-like homologs. After the identification of serpin-homolog raw reads in *C. shasta* and *M. pendula* transcriptomes (using local blast search, respectively), serpin sequences were assembled in Geneious Prime 2019 (Biomatters Ltd., Auckland, New Zealand) [34] (Dataset S1). The serpin conserved domain, named signature was detected using its consensus pattern: [LIVMFY]-{G}-[LIVMFYAC]-[DNQ]-[RKHQS]-[PST]–F-[LIVMFY]-[LIVMFYC]-x-[LIVMFAH]; PROSITE entry PS00284). To identify serpins and predict their function, online searches were conducted in the Merops-MPEP peptidase database Release 11.1, and Pfam (by EMBL-EBI), and biosequence analysis was also performed using HMMER [35,36,37]. Myxozoan serpins were classified to functional groups (types) using the UniProt BLAST with the UniProtKB reference proteomes plus the Swiss-Prot target database and Merops-MPEP [35,38].

### 2.2. PCR Verification of in Silico Identified Myxozoan Serpins

The triactinomyxon-type actinospores (TAMs) of *M. cerebralis* (previously characterized by Sipos et al [39]) and *M. pseudodispar* lineage GER (NCBI Acc. No. EF466088) were harvested from the life cycles maintained in the Fish Parasitology laboratory of the Institute for Veterinary Medical Research, Centre for Agricultural Research, Budapest, Hungary, as described previously by Eszterbauer et al. [40]. The identities of parasite TAMs were confirmed using species-specific 18S rDNA PCR and DNA sequencing, according to Sipos et al. [39]. *S. molnari* proliferative, presporogonic blood stages were collected from the laboratory lineage maintained by intraperitoneal infection of parasite blood stages into specific pathogen-free common carp (*Cyprinus carpio*) in the Laboratory of Fish Protistology of the Institute of Parasitology, Biology Centre of CAS, Ceske Budejovice, Czech Republic [41]. *M. lieberkuehni* sporogonic stages were collected from the urinary bladder of pike (*Esox lucius*) in Třeboň area, Czech Republic. Contaminating myxozoans were not detected in any sample.

The sequences of *M. cerebralis, M. pseudodispar, S. molnari*, and *M. lieberkuehni* serpins which could not be retrieved from public databases, were obtained using serpin-specific PCR assays. As only partial *M. cerebralis* serpin sequences without signature could be extracted from public databases, the sequence of five *M. cerebralis* serpins (i.e., Mc-S1, S3, S4, S5, and S6) was complemented with rapid amplification of cDNA ends (RACE PCR). The procedures of nucleic acid extractions (both RNA and DNA), cDNA synthesis, PCR, 5’/3’ RACE and DNA sequencing including the list of oligonucleotides are described in the supplementary material (Table S2).

### 2.3. Sequence Alignment and Pairwise Sequence Identity Calculations

Tree inferences and the pairwise alignment-based sequence identity analysis were conducted on aa sequences of serpin genes or transcripts. Host contaminants—mainly fish serpins—identified in the examined databases were excluded from the final dataset, on the basis of phylogenetic analysis (Table S1). Similarly, sequences without the conserved domain signature were also excluded from further analyses, as well as the too distant group of putative Kazal-type serpins and sequences having multiple signature domains. Besides myxozoan serpins, protist and animal serpin representatives were included into the analysis (Table S1).

Multiple alignments were conducted using MAFFT [42] with the E-INS-i algorithm, optimized for multiple conserved domains and long gaps. Alignments were edited manually to remove non-homologous regions. To compare the conserved serpin domains (i.e., motif and signature) among taxa and serpin types, the sequences of the motif, the signature, and the reactive site loop in between, were extracted from the sequences in the final alignment and aligned with MAFFT (E-INS-i algorithm) after sorting by taxa and serpin type, respectively. The pairwise sequence identity analysis was conducted using the G-INS-I alignment algorithm of MAFFT in the Sequence Demarcation Tool v1.2 [43]. Cut-off values (29% and 95%) were set on the basis of the calculated sequence identity values. The identity scores were calculated as 1-(M/N) where M is the number of mismatching residues and N is the total number of positions along the alignment, where none of the sequences has a gap character [43]. 

### 2.4. Phylogenetic Analysis

For tree inference, first a broad-range phylogenetic analysis was conducted, then only myxozoan serpins were analyzed from the previous dataset. For both tree inferences, the best evolutionary model was predicted using ModelTest-NG v0.1.5 [44], and was shown to be the Le-Gascuel aa replacement matrix with 4 categories of gamma-distributed rate heterogeneity [45]. Moreover, the proportion of the invariant sites (LG+I+Γ4) was also taken into account. Both tree inferences were conducted using RAxML-NG v0.9.0 [46]. The robustness of the trees was determined with non-parametric, transfer bootstrap expectation (TBE) calculation using 1,000 repeats. Phylogenetic trees were visualized using MEGA 7 [47] and edited using Inkscape v 0.92.4.

## 3. Results

### 3.1. Serpin Screening

Altogether 224 serpin-like sequences from 71 species ranging from protists to vertebrates were selected and compared (Table S1). The number of serpin-like proteins identified in myxozoan genomes and transcriptomes was 146 and varied considerably by species. The highest number of serpin-like proteins (114) was detected in the *T. kitauei* genome. Of these 114, we found 26 serpins present in the transcriptome of fish-dwelling *T. kitauei* myxospores (Table S1). Among others, alpha1-antitrypsin-like, alpha1-antichymotrypsin-like, and protein Z-dependent protease-like inhibitors (clade A), leukocyte elastase inhibitor-like (clade B), antithrombin-like (clade C), nexin-like serpins (clade E) and myoepithelium derived serpin-like homologs (clade I) were detected in the transcriptome (Table 1).

Twenty-eight previously undescribed myxozoan serpin homologs were identified in the examined databases. Compared to *T. kitauei*, notably lower number of serpins was distinguished in *M. cerebralis* (nine sequences of seven serpin homologs), *M. pendula* (six sequences of five homologs), *M. pseudodispar* (two), *M. squamalis* (four; of which three were newly described), *S. molnari* (two), *C. shasta* (three), *H. salminicola* (three), *M. lieberkuehni* (one), and *K. iwatai* (two) (Table 1 and S1). Serpin-like proteins were not detected in the partial genome of *Enteromyxum leei* and *Sphaeromyxa zaharoni*. Altogether ten new serpin sequences of the myxozoan species *M. cerebralis*, *M. pseudodispar*, *S. molnari* and *M. lieberkuehni*, confirmed with PCR and sequencing, were submitted to NCBI GenBank (accession Nos. are listed in Table S1).

### 3.2. Serpin Diversity

**Figure 1:**
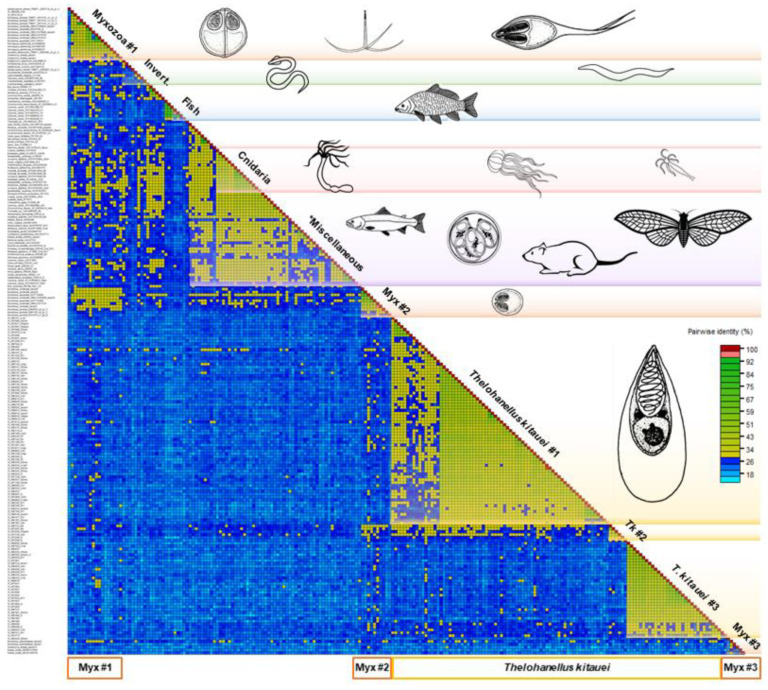
Pairwise sequence identity matrix of 224 serpin sequences using Sequence Demarcation Tool v1.2. Cut-off values: 29% and 95%. Blue squares represent sequence identity below 29%, green squares 29–94%, red ≥ 95%. The length of the protein sequences varied between 127 and 518 amino acid. Myxozoan groups #1, #2, and #3 contain a variety of myxozoan serpins (*Myxobolus cerebralis, M. pendula, M. pseudodispar, M. squamalis, Ceratonova shasta, Sphaerospora molnari, Myxidium lieberkuehni, Kudoa iwatai, Henneguya salminicola*), except the ones of *Thelohanellus kitauei*. *Miscellaneous: sequence identity group composed of protists, invertebrates, and vertebrates.

High intra- and interspecific variability was detected among the 224 serpins examined. The average aa sequence identity was 25–30%, but identities as low as 10% were not exceptional either (Figure 1). 

Amongst serpins of myxozoans, the sequence identity varied between 12 and 98%. They clustered into three subgroups, however, identity values between and within Myxozoa group #1 and #2 did not differ notably. The group Myxozoa #3, composed of *K. iwatai*, *M. pseudodispar* serpins and *C. shasta*-S3, showed <20% sequence identity to each other and to the rest of serpins (Figure 1). *T. kitauei* serpins formed two large and one small sequence identity group in the matrix, and clearly diverged from other myxozoan serpins, with the exception of two *T. kitauei* serpins, which grouped with Myxozoa #1 (Figure 1). Free-living cnidarians clustered together, but located within the Miscellaneous group composed of protists, invertebrates, and vertebrates, showing 28–43% identity within the group. The fish-derived H1-type (i.e., heat shock protein 47-like) serpins formed a distinct group in the matrix.

### 3.3. Conserved Domains

**Figure 2:**
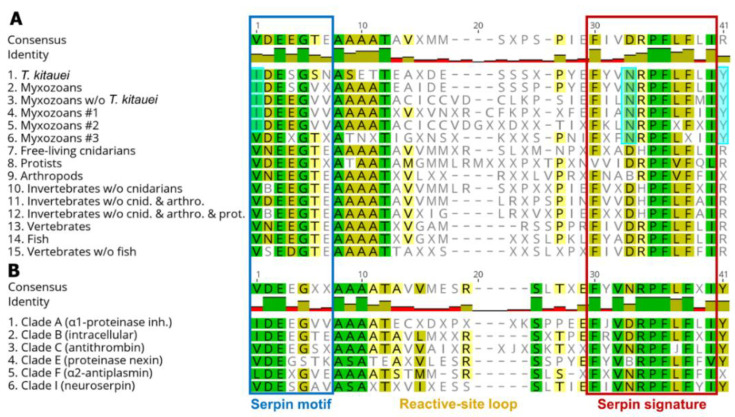
Comparison of consensus amino acid sequences of conserved serpin domains, motif and signature, sorted by (**A**) taxon, and (**B**) serpin type classified in clades. Amino acid positions, which differ in myxozoans compared to other taxa, were highlighted pale blue.

The conserved serpin domains showed taxon-specific differences. For myxozoans, amino acids at certain positions (alignment pos. 1, 33, and 41) in the conserved motifs and signatures were consistently different compared to other taxa (Figure 2A; Dataset S2). The consensus motif sequence of myxozoan clade #3 was an exception that showed similar pattern to non-myxozoan taxa (Figure 2A, line 6). 

The comparison of serpin types has shown that the consensus aa sequences were rather uniform among groups. Serpins belonging to serpin clades A and B, which were the dominant serpin types among myxozoans, differed in a couple of aa positions (i.e., alignment pos. 1—in motif, and pos. 33—in signature) from other serpin clades (Figure 2B; Dataset S2).

### 3.4. Serpin Phylogeny

**Figure 3:**
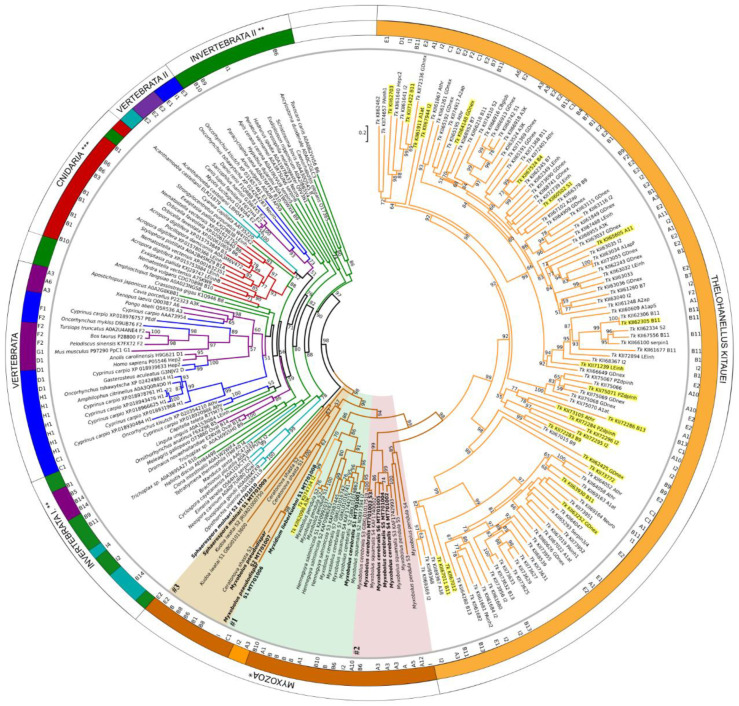
Maximum-likelihood phylogenetic tree inference of serpin genes, based on a 423-aa-long alignment of 224 protein sequences (RAxML, evolutionary model LG+I+Γ4, 1000 bootstrap replicates). Serpin sequences are represented by species name and GenBank or Uniprot accession numbers. Serpin types, where determined (see Table 1 and Table S1), are indicated in the inner ring. The tree was rooted on the midpoint, and bootstrap values are given as percentages. Tree branches were colored according to taxonomy: vertebrate (purple), fish (blue), protists (turquoise), invertebrate (green), free-living Cnidaria (red), Myxozoa (brown), *Thelohanellus kitauei*, Tk (orange). * w/o *T. kitauei*, ** w/o Cnidaria and protists; *** w/o Myxozoa. Myxozoan subclades #1, #2, and #3 were highlighted with background color (#1: green, #2: red, #3: beige). Newly obtained serpin sequences were labelled bold. *T. kitauei* serpins, which were present in the transcriptome obtained from fish-infecting myxospore, were highlighted yellow.

Maximum-likelihood phylogenetic tree inference of 224 serpins showed clear separation of myxozoan serpins from other taxa (Figure 3; Figure S1; Dataset S3).

Serpin sequences clustered rather based on taxonomy and not on serpin types, but in many cases the support of the branches was low. Surprisingly, the majority of *T. kitauei* serpins clustered separately from other myxozoans, although sister to them, and they composed two subclades with high bootstrap support. Both subclades contained various functional types, from Clade A to I. Two *T. kitauei* serpins, a myoepithelium-derived serpin (I2; KII74734) and an alpha1-antichymotrypsin (A3; KII62098) were grouped separately from the large group, within the clade of Myxozoa. Besides, three serpins of *M. pendula* were located within *T. kitauei* subclade II. The rest of myxozoans were located as a sister clade of *T. kitauei*, and formed three subclades (#1, #2 and #3). *S. molnari*-S2 clustered with a blood fluke, *Opistorchis viverrini,* however with low bootstrap support (Figure 3).

Interestingly, none of the myxozoan serpins were grouped with any of their free-living cnidarian relatives. Free-living cnidarian serpins were located close to other invertebrate taxa, distantly from myxozoan groups. Vertebrate taxa (including fish) formed two main clusters close to invertebrates (Figure 3). A tendency to cluster according to the type of serpins was not observed.

**Figure 4:**
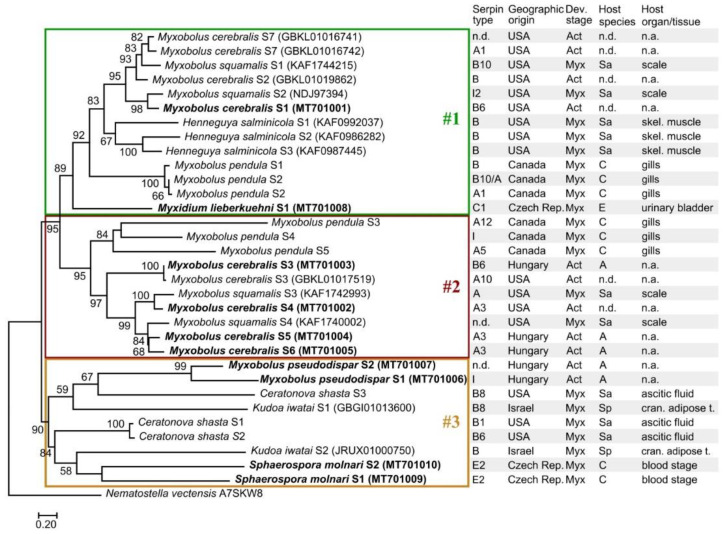
Maximum-likelihood tree of 32 myxozoan serpin sequences, on the basis of a 365-long amino acid sequence alignment (RAxML, LG+I+Γ4 model, 1000 bootstrap replicates). The serpins of *Thelohanellus kitauei* were not included in the analysis. The free-living Cnidaria, *Nematostella vectensis* was used as outgroup. Bootstrap values are given as percentages. Sequences obtained with specific PCR amplification followed by DNA sequencing are highlighted bold. Subclades were indicated with boxes #1, #2, and #3. Developmental stages: Myx—myxospore; Act—actinospore; Host species: Sa—Salmonidae; C—Cyprinidae; A—Annelida; E—Esocidae; Sp—Sparidae. n.d.—no data; n.a.—not applied.

Myxozoan serpins formed three subclades in their phylogenetic reconstruction (Figure 4; Dataset S4). Despite the high genetic diversity of serpins, the tree topology was in correspondence with the findings based on aa sequence identity matrix except for *C. shasta* serpins of the subclade #3, which were located in subclade #1 in the sequence identity matrix (Figure 1). The myxozoan subclades #1, #2, and #3 in Figure 4 were composed of the same serpins as the subclades in Figure 3. The myxozoan subclade #1 included *M. cerebralis* S4–S6, three *M. pendula* serpins, two *M. squamalis* serpins, and the basal *M. lieberkuehni* serpin. Most serpins in this subclade were identified as intracellular types (clade B). The subclade #2 contained the rest of *M. cerebralis*, *M. pendula*, and *M. squamalis* serpins, most of which were predicted as clade A type (i.e., alpha1-proteinase inhibitor). Although the subclade #3 was highly supported, the location of serpins within the group was rather uncertain. The serpins identified in *M. pseudodispar*, *K. iwatai*, *C. shasta*, and *S. molnari* formed the genetically most diverse subgroup among myxozoan serpins.

## 4. Discussion

In the present study, we analyzed the phylogenetic relationships of 146 myxozoan serpins, and compared them to those of other organisms. Previous studies concluded that the elucidation of phylogenetic relationships among animal serpins poses difficulties due to the high genetic variabilities [9,48]. Serpin genes represent a substantial fraction of metazoan genomes. Multiple expansions of serpins may have occurred that resulted in numerous paralogs, whereas in other lineages, such as fungi, serpins seem to be rare [48]. 

Our results suggest that lower number of serpins is present in the genome of *T. kitauei* than it was previously reported (114 vs. 182) [27]. Of the 182, we detected fish contaminants, and identical serpin sequences, which were excluded from the analysis. Furthermore, partial sequences lacking conserved domains could not be identified as serpins with certainty, thus 114 sequences remained in the analysis. The freshwater, histozoic species *T. kitauei* is responsible for intestinal giant cystic disease in common carp, *Cyprinus carpio*. The clinical signs include thinned intestinal wall, ascites in the abdominal cavity, and edema in the serosal layer of the intestine. The numerous parasite plasmodia cause swellings and often block the intestinal lumen of affected fish, thus the infection may result in high mortality. Infection triggers heavy cellular immune reactions in fish, with elevated level of TNF-alpha, IL-1beta, IL-6, and IgM [49]. To overcome the host reaction, the parasite may need an extensive immunomodulatory strategy for successful invasion, dissemination, and development. The wide variety of serpins identified in *T. kitauei* may serve as efficient mediators for this purpose. Our finding that alpha1-antitrypsins and leukocyte elastase inhibitors are present in the transcriptome supports this hypothesis. However, it still remains unclear why *T. kitauei* serpins are so divergent from the ones of other myxozoan species, and why they formed a distinct phylogenetic group. It is likely that *T. kitauei* requires serpins for purposes unusual for other myxozoans. This may result in the presence of a wide range of serpin families, as the parasite enters fish via the digestive tract and developmental stages are exposed to a number of digestive enzymes from the host during invasion. Besides, the antithrombins and protein Z-dependent protease inhibitors, which are involved in resolving blood coagules [50,51] and are both present in the transcriptome, are likely helping the parasite to exit blood vessels or feed on e.g., red blood cells.

Serpins were not found in the genome of marine myxozoans *E. leei* infecting the intestinal epithelium of the gilt-head sea bream (*Sparus aurata*), and in *S. zaharoni* forming plasmodia in the gall bladder of the devil firefish (*Pterois miles*). *Enteromyxum* spp., especially *E. leei*, often cause notable damage in the intestine of affected fish that is accompanied by intense immune response [52,53], therefore we expected the presence of various serpins in the genome, similarly to *T. kitauei*. As the genome of *E. leei* and *S. zaharoni* is still partial, it is likely that protease inhibitors will be identified when their complete genome becomes available. Despite myxozoans having extremely reduced genomes, they still have maintained or even diversified their serpin sets, hence these molecules must be important for parasite survival in the host. On the other hand we must add that technical difficulties caused by introns and associated frame shift in open reading frames may also hamper the retrieval of serpins from non-annotated genomes. 

Host environment remarkably influences the evolution of myxozoan parasites [54]. Whether there is any correlation between the marine or freshwater environment and the phylogenetic position of serpins, conclusions cannot be drawn based on our current knowledge, as almost all serpins were identified in freshwater myxozoan species. The sole exception was *K. iwatai*, which forms plasmodia in the intracranial adipose tissue of gilt-head sea bream. The two serpins identified in *K. iwatai* clustered in the genetically rather diverse myxozoan subclade #3. The phylogenetic gaps may be filled by serpins identified in future. With higher number of marine representatives, the phylogenetic position of serpins from marine parasites, and thereby the relevance of the host environment can be examined.

Although exceptions occurred, myxozoan serpins grouped mainly according to taxonomy. This was in accordance with the findings of Irving et al. [9] regarding the phylogenetic analysis of non-vertebrate serpins. The genetic differences among *C. shasta* serpins detected by Alama-Bermejo et al. [32] were confirmed in our study. Although all three analyzed serpins were putatively intracellular (clade B), Cs-S3 were located far from Cs-S1 & Cs-S2 on the phylogenetic tree, potentially indicating an independent origin. The *Myxobolus* and *Henneguya* serpins, represented in the highest number of homologs, grouped mainly together. The serpins of *M. cerebralis*, the cause of whirling disease in salmonids, seem to be in close relation to the serpins of *M. squamalis* forming plasmodia in the scales of salmonids, *M. pendula* from the gills of creek chub, *Semotilus atromaculatus*, and *H. salminicola* from the skeletal muscle of salmonids.

Most of the serpins detected in Myxozoa putatively belong to two serpin types, the extracellular, alpha1-antitrypsin and alpha1-antiproteinase (clade A) and the intracellular serpins (clade B). Serpins of clade A inhibit various proteases (not merely trypsin), and their main function is to protect tissues from enzymes of inflammatory cells. Intracellular serpins may have various functions, from the inhibition of anti-coagulatory proteases to enhancing lysosome stability [19,55]. Intracellular serpins were detected in the excretory product of blood-feeding parasites such as ticks [13,14], or in the saliva of the fish ectoparasite arthropod, *Argulus foliaceus* [56]. These studies concluded that intracellular serpins are supposed to be one of the major components involved in evasion of the host defense mechanisms for successful feeding. Our current knowledge about the function of myxozoan serpins is scarce. The effect of serpin type on their phylogenetic placement was recognizable but not prominent, by forming subclades #1 and #2 within Myxozoa. Two serpins detected in *M. pseudodispar*, the skeletal muscle parasite of cyprinids, and another two from *S. molnari*, the gill parasite of common carp, were exceptions, as they neither grouped with other members of Myxobolidae, nor belonged to serpin clade A or B. The function of myxozoan serpins was predicted based on translated protein sequences only. However, it is likely that serpins may be involved in anti-inflammatory and anti-coagulatory processes during host invasion [32]. Previous study concluded that the phylogenetic clustering according to serpin type is more prominent for vertebrates [9]. Although only limited number of non-myxozoan serpin representatives was involved in our analysis, fish serpins were present in relatively high number, and they clearly grouped by serpin type, as the extra- and the intracellular serpins, and the heat-shock proteins of fish located distantly on the tree.

Surprisingly, serpins of free-living cnidarians showed distant relation to the ones of their parasitic relatives, the myxozoans. Current knowledge about the function and characteristics of protease inhibitors of early metazoans is rather limited [57,58]. In the large-scale gene linkage study, Putnam et al. [57] found genome complexity and novel genes enriched for animal function when comparing the genome of the sea anemone, *Nematostella vectensis*, to that of bilaterians. Jellypin (JP), a serpin identified in jellyfish, *Cyanea capillata* is a putative antichymotrypsin-like serpin (clade A3), whereas its structural elements and phylogenetic analysis showed close relation to intracellular serpins (clade B) [58]. These findings suggested that JP was one of the oldest metazoan serpins to be identified and arose around the divergence between plants and animals, ∼1000 MYA. Furthermore, they predicted that JP may have a unique, yet unknown function. However, our findings suggest that myxozoan serpins do not have a cnidarian origin. Maybe the genes encoding serpins common in free-living cnidarians were lost due to the massive genome reduction, when myxozoans became parasites of invertebrate hosts. At a later stage of evolution, when myxozoans invaded vertebrate hosts, it is possible that they acquired serpin-coding genes from fish parasite protists, nematodes, or flukes. This would explain why the liver fluke *Opisthorchis viverrini*, the second intermediate host of which is fish, clustered with *S. molnari* S2, in the sister group of myxozoan serpins. This phenomenon would not be exceptional for parasites. A previous study suggested that unicellular human pathogens such as *Toxoplasma gondii*, *Eimeria tenella*, and *Giardia lamblia* acquired serpins probably from multicellular organisms via horizontal gene transfer [59]. However, the long branch attraction and the well-known technical issue of phylogenetic tree reconstruction might also be responsible for the seemingly close relationship between *S. molnari*-S2 and the serpin of the liver fluke. Besides genome reduction and horizontal gene transfer, the diversification by gene modification could also explain the genetic distinction of myxozoan serpins. This idea is supported by the strongly derived character of other myxozoan genes, even ones with very conservative character such as actin [6].

Serpins are thought to share a highly ordered tertiary structure [10,60]. The conserved domains, motif, and signature, are near the C-terminus. Between them, a small functional domain called the reactive-site loop is located that is exposed at the surface of the protein. The aa sequence of the reactive-site loop is hypervariable among animal serpins, and seems to be responsible for the dramatic changes in the proteinase selectivity of a serpin [60]. We also detected taxon-related alterations in the conserved domains when compared myxozoan serpins to those of other taxa. Although functionally low variance was predicted, as the majority of serpins was typed as clade A and B, sequence alterations were observable, and certain aa residues were exclusive characters of Myxozoa. As previous findings highlight the roles of conserved domains and residues in the mechanism of conformational changes, and thereby the functional variability of these proteins [9], further studies are in progress to elucidate the structural and functional characteristics of the newly discovered myxozoan serpins.

## 5. Conclusions

Our findings suggest that serpins are genetically rather diverse, just like they are putatively variable in function. Although the high genetic variability makes the reconstruction of their phylogenetic position difficult, the distinct localization of myxozoan serpins from those of other taxa highlights their unique origin, and may indicate novel protein functions in this parasitic group.

## Figures and Tables

**Table 1:** Examined myxozoan serpins, classified according to Gettins [19]. The typing was conducted using UniProt BLAST and Merops-MPEP. Further details are available in Table S1. GenBank accession numbers are after serpin names in parenthesis. Bold: newly identified serpin-homologs; bold-italics: new sequence additions to GenBank.

Clade Identifier	Clade Name	Predicted Representatives among Myxozoans
A	alpha1-proteinase inhibitor (antitrypsin-like, extracellular)	***Mc-S4 (MT701002)*; *Mc-S5 (MT701004)*; *Mc-S6 (MT701005)*; Mc-S7 (GBKL01016742); Mpe-S2 (SRX1269149); Mpe-S3 (SRX1269149); Mpe-S5 (SRX1269149); Ms-S3 (KAF1742993);** Tk (various; see in Table S1)
B	intracellular; ov-serpins	***Mc-S1 (MT701001)*; Mc-S2 (GBKL01019862); *Mc-S3 (MT701003)*; Mpe-S1 (SRX1269149); Ms-S1 (KAF1744215); Cs-S1 (SRX3741971); Cs-S2 (SRX3741971); Cs-S3 (SRX3741971); Hs-S1 (KAF0992037); Hs-S2 (KAF0986282); Hs-S3 (KAF0987445); Ki-S1 (GBGI01013600); Ki-S2 (JRUX01000750);** Tk (various; see in Table S1)
C	antithrombin	***Ml-S1 (MT701008)*;** Tk-(KII73105); Tk-(KII72401); Tk-(KII65195); Tk-(KII64058); Tk-(KII64052); Tk-(KII61867)
D	heparin cofactor 2	Tk-(KII61640)
E	proteinase nexin	***Sm-S1 (MT701009)*; *Sm-S2 (MT701010)*;** Tk (various)
F	alpha2-antiplasmin	Tk-(KII74917); Tk-(KII67102); Tk-(KII63034); Tk-(KII61248)
I	neuroserpin	***Mps-S1 (MT701006)*;** Ms-S2 (NDJ97394); **Mpe-S4 (SRX1269149);** Tk (various; see in Table S1)
-	unknown	***Mps-S2 (MT701007)***; **Ms-S4 (KAF1740002)**

Mc: *Myxobolus cerebralis*; Mpe: *Myxobolus pendula*; M*ps: *Myxobolus pseudodispar*; Ms: *Myxobolus squamalis; Sm: *Sphaerospora molnari*; Cs: *Ceratonova shasta*; Hs: *Henneguya salminicola*; Ml: *Myxidium lieberkuehni*; Ki: *Kudoa iwatai*; Tk: *Thelohanellus kitauei*. S: serpin.

## Supplementary Materials

The following are available online at https://www.mdpi.com/2076-2607/8/10/1502/s1.

## References

[1] Okamura B., Gruhl A., Bartholomew J.L. (2015). Myxozoan Evolution, Ecology and Development.

[2] Forró B., Eszterbauer E. (2016). Correlation between host specificity and genetic diversity for the muscle-dwelling fish parasite *Myxobolus pseudodispar*: Examples of myxozoan host-shift?. Folia Parasitol..

[3] Holzer A.S., Bartošová-Sojková P., Born-Torrijos A., Lövy A., Hartigan A., Fiala I. (2018). The joint evolution of the Myxozoa and their alternate hosts: A cnidarian recipe for success and vast biodiversity. Mol. Ecol..

[4] Lisnerová M., Fiala I., Cantatore D., Irigoitia M., Timi J., Pecková H., Bartošová-Sojková P., Sandoval C.M., Luer C., Morris J. (2020). Mechanisms and Drivers for the Establishment of Life Cycle Complexity in Myxozoan Parasites. Biology.

[5] Eszterbauer E., Kallert D.M., Grabner D., El-Matbouli M. (2009). Differentially expressed parasite genes involved in host recognition and invasion of the triactinomyxon stage of *Myxobolus cerebralis* (Myxozoa). Parasitology.

[6] Hartigan A., Estensoro I., Vancová M., Bílý T., Patra S., Eszterbauer E., Holzer A.S. (2016). New cell motility model observed in parasitic cnidarian *Sphaerospora molnari* (Myxozoa:Myxosporea) blood stages in fish. Sci. Rep..

[7] Feist S.W., Morris D.J., Alama-Bermejo G., Holzer A.S. (2015). Cellular Processes in Myxozoans. Myxozoan Evolution, Ecology and Development.

[8] Alama-Bermejo G., Holzer A.S., Bartholomew J.L. (2019). Myxozoan Adhesion and Virulence: *Ceratonova shasta* on the Move. Microorganisms.

[9] Irving J.A., Pike R.N., Lesk A.M., Whisstock J.C. (2000). Phylogeny of the Serpin Superfamily: Implications of Patterns of Amino Acid Conservation for Structure and Function. Genome Res..

[10] Silverman G.A., Bird P.I., Carrell R.W., Church F.C., Coughlin P.B., Gettins P.G.W., Irving J.A., Lomas D.A., Luke C.J., Moyer R.W. (2001). The serpins are an expanding superfamily of structurally similar but functionally diverse proteins. Evolution, mechanism of inhibition, novel functions, and a revised nomenclature. J. Biol. Chem..

[11] Mulenga A., Sugino M., Nakajima M., Sugimoto C., Onuma M. (2001). Tick-encoded serine proteinase inhibitors (Serpins); Potential target antigens for tick vaccine development. J. Vet. Med. Sci..

[12] Xu T., Lew-Tabor A., Rodriguez-Valle M. (2016). Effective inhibition of thrombin by *Rhipicephalus microplus* serpin-15 (RmS-15) obtained in the yeast Pichia pastoris. Ticks Tick Borne Dis.

[13] Tirloni L., Reck J., Terra R.M.S., Martins J.R., Mulenga A., Sherman N.E., Fox J.W., Yates J.R., Termignoni C., Pinto A.F.M. (2014). Proteomic Analysis of Cattle Tick *Rhipicephalus (Boophilus) microplus* Saliva: A Comparison between Partially and Fully Engorged Females. PLoS ONE.

[14] Tirloni L., Kim T.K., Coutinho M.L., Ali A., Seixas A., Termignoni C., Mulenga A., da Silva Vaz I. (2016). The putative role of *Rhipicephalus microplus* salivary serpins in the tick-host relationship. Insect Biochem. Mol. Biol..

[15] Modica M.V., Sunagar K., Holford M., Dutertre S. (2020). Editorial: Diversity and Evolution of Animal Venoms: Neglected Targets, Ecological Interactions, Future Perspectives. Front. Ecol. Evol..

[16] Schick C., Pemberton P.A., Shi G.P., Kamachi Y., Çataltepe S., Bartuski A.J., Gornstein E.R., Brömme D., Chapman H.A., Silverman G.A. (1998). Cross-class inhibition of the cysteine proteinases cathepsins K, L, and S by the serpin squamous cell carcinoma antigen 1: A kinetic analysis. Biochemistry.

[17] Huntington J.A., Read R.J., Carrell R.W. (2000). Structure of a serpin-protease complex shows inhibition by deformation. Nature.

[18] Law R.H.P., Zhang Q., McGowan S., Buckle A.M., Silverman G.A., Wong W., Rosado C.J., Langendorf C.G., Pike R.N., Bird P.I. (2006). An overview of the serpin superfamily. Genome Biol..

[19] Gettins P.G.W. (2002). Serpin Structure, Mechanism, and Function. Chem. Rev..

[20] Quezada L.A.L., McKerrow J.H. (2011). Schistosome serine protease inhibitors: Parasite defense or homeostasis?. An. Acad. Bras. Cienc..

[21] Valdivieso E., Perteguer M.J., Hurtado C., Campioli P., Rodriguez E., Saborido A., Martinez-Sernandez V., Gomez-Puertas P., Ubeira F.M., Garate T. (2015). ANISERP: a new serpin from the parasite Anisakis simplex. Parasit. Vectors.

[22] Maizels R.M., Gomez-Escobar N., Gregory W.F., Murray J., Zang X.X. (2001). Immune evasion genes from filarial nematodes. Int. J. Parasitol..

[23] Toubarro D., Lucena-Robles M., Nascimento G., Santos R., Montiel R., Veríssimo P., Pires E., Faro C., Coelho A.V., Simões N. (2010). Serine protease-mediated host invasion by the parasitic nematode Steinernema carpocapsae. J. Biol. Chem..

[24] Doyle P.S., Zhou Y.M., Hsieh I., Greenbaum D.C., McKerrow J.H., Engel J.C. (2011). The trypanosoma cruzi protease cruzain mediates immune evasion. PLoS Pathog..

[25] Faria M.S., Reis F.C., Azevedo-Pereira R.L., Morrison L.S., Mottram J.C., Lima A.P. (2011). Leishmania inhibitor of serine peptidase 2 prevents TLR4 activation by neutrophil elastase promoting parasite survival in murine macrophages. J. Immunol..

[26] Alam A., Bhatnagar R.K., Relan U., Mukherjee P., Chauhan V.S. (2013). Proteolytic activity of Plasmodium falciparum subtilisin-like protease 3 on parasite profilin, a multifunctional protein. Mol. Biochem. Parasitol..

[27] Yang Y., Xiong J., Zhou Z., Huo F., Miao W., Ran C., Liu Y., Zhang J., Feng J., Wang M. (2014). The genome of the myxosporean *Thelohanellus kitauei* shows adaptations to nutrient acquisition within its fish host. Genome Biol. Evol..

[28] Chang E.S., Neuhof M., Rubinstein N.D., Diamant A., Philippe H., Huchon D., Cartwright P. (2015). Genomic insights into the evolutionary origin of Myxozoa within Cnidaria. Proc. Natl. Acad. Sci. USA.

[29] Yahalomi D., Atkinson S.D., Neuhof M., Sally Chang E., Philippe H., Cartwright P., Bartholomew J.L., Huchon D. (2020). A cnidarian parasite of salmon (Myxozoa: *Henneguya*) lacks a mitochondrial genome. Proc. Natl. Acad. Sci. USA.

[30] Foox J., Ringuette M., Desser S.S., Siddall M.E. (2015). In silico hybridization enables transcriptomic illumination of the nature and evolution of Myxozoa. BMC Genom..

[31] Nesnidal M.P., Helmkampf M., Bruchhaus I., El-Matbouli M., Hausdorf B. (2013). Agent of whirling disease meets orphan worm: phylogenomic analyses firmly place Myxozoa in Cnidaria. PLoS ONE.

[32] Alama-Bermejo G., Meyer E., Atkinson S.D., Holzer A.S., Wiśniewska M.M., Kolísko M., Bartholomew J.L. (2020). Transcriptome-wide comparisons and virulence gene polymorphisms of host-associated genotypes of the cnidarian parasite *Ceratonova shasta* in salmonids. Genome Biol. Evol..

[33] Hartigan A., Kosakyan A., Pecková H., Eszterbauer E., Holzer A.S. (2020). Transcriptome of *Sphaerospora molnari* (Cnidaria, Myxosporea) blood stages provides proteolytic arsenal as potential therapeutic targets against sphaerosporosis in common carp. BMC Genom..

[34] Altschul S.F., Madden T.L., Schaffer A.A., Zhang J.H., Zhang Z., Miller W., Lipman D.J. (1997). Gapped BLAST and PSI-BLAST: a new generation of protein database search programs. Nucleic Acids Res..

[35] Rawlings N.D., Barrett A.J., Thomas P.D., Huang X., Bateman A., Finn R.D. (2018). The MEROPS database of proteolytic enzymes, their substrates and inhibitors in 2017 and a comparison with peptidases in the PANTHER database. Nucleic Acids Res..

[36] El-Gebali S., Mistry J., Bateman A., Eddy S.R., Luciani A., Potter S.C., Qureshi M., Richardson L.J., Salazar G.A., Smart A. (2019). The Pfam protein families database in 2019. Nucleic Acids Res..

[37] Finn R.D., Clements J., Eddy S.R. (2011). HMMER web server: Interactive sequence similarity searching. Nucleic Acids Res..

[38] Bateman A. (2019). UniProt: A worldwide hub of protein knowledge. Nucleic Acids Res..

[39] Sipos D., Ursu K., Dán Á., Herczeg D., Eszterbauer E. (2018). Susceptibility-related differences in the quantity of developmental stages of *Myxobolus* spp. (Myxozoa) in fish blood. PLoS ONE.

[40] Eszterbauer E., Atkinson S., Diamant A., Morris D., El-Matbouli Mansour M., Hartikainen H. (2015). Myxozoan life cycles: Practical approaches and insights. Myxozoan Evolution, Ecology and Development.

[41] Korytář T., Wiegertjes G.F., Zusková E., Tomanová A., Lisnerová M., Patra S., Sieranski V., Šíma R., Born-Torrijos A., Wentzel A.S. (2019). The kinetics of cellular and humoral immune responses of common carp to presporogonic development of the myxozoan *Sphaerospora molnari*. Parasit. Vectors.

[42] Katoh K., Standley D.M. (2013). MAFFT multiple sequence alignment software version 7: improvements in performance and usability. Mol. Biol. Evol..

[43] Muhire B.M., Varsani A., Martin D.P. (2014). SDT: a virus classification tool based on pairwise sequence alignment and identity calculation. PLoS ONE.

[44] Darriba D., Posada D., Kozlov A.M., Stamatakis A., Morel B., Flouri T. (2020). ModelTest-NG: a new and scalable tool for the selection of DNA and protein evolutionary models. Mol. Biol. Evol..

[45] Le S.Q., Gascuel O. (2008). An improved general amino acid replacement matrix. Mol. Biol. Evol..

[46] Kozlov A.M., Darriba D., Flouri T., Morel B., Stamatakis A. (2019). RAxML-NG: A fast, scalable, and user-friendly tool for maximum likelihood phylogenetic inference. Bioinformatics.

[47] Kumar S., Stecher G., Tamura K. (2016). MEGA7: Molecular Evolutionary Genetics Analysis version 7.0 for bigger datasets. Mol. Biol. Evol..

[48] Kumar A., Ragg H. (2008). Ancestry and evolution of a secretory pathway serpin. BMC Evol. Biol..

[49] Liu T., Wei W.Y., Wang K.Y., Yang Q., Wang E.L. (2019). Pathological and immunological analyses of *Thelohanellus kitauei* (Myxozoa:Myxosporea) infection in the scattered mirror carp, *Cyprinus carpio*. Sci. Rep..

[50] Jordan R.E. (1983). Antithrombin in vertebrate species: Conservation of the heparin-dependent anticoagulant mechanism. Arch. Biochem. Biophys..

[51] Han X., Fiehler R., Broze G.J. (1998). Isolation of a protein Z-dependent plasma protease inhibitor. Proc. Natl. Acad. Sci. USA.

[52] Sitjà-Bobadilla A., Calduch-Giner J., Saera-Vila A., Palenzuela O., Álvarez-Pellitero P., Pérez-Sánchez J. (2008). Chronic exposure to the parasite *Enteromyxum leei* (Myxozoa: Myxosporea) modulates the immune response and the expression of growth, redox and immune relevant genes in gilthead sea bream, *Sparus aurata* L.. Fish Shellfish Immunol..

[53] Sitjà-Bobadilla A., Schmidt-Posthaus H., Wahli T., Holland J.W., Secombes C.J. (2015). Fish Immune Responses to Myxozoa. Myxozoan Evolution, Ecology and Development.

[54] Fiala I., Bartosova P. (2010). History of myxozoan character evolution on the basis of rDNA and EF-2 data. BMC Evol. Biol..

[55] Marciniak S.J., Lomas D.A. (2008). Intracellular serpins, firewalls and tissue necrosis. Trends Cell Biol..

[56] AmbuAli A., Monaghan S.J., McLean K., Inglis N.F., Bekaert M., Wehner S., Bron J.E. (2020). Identification of proteins from the secretory/excretory products (SEPs) of the branchiuran ectoparasite *Argulus foliaceus* (Linnaeus, 1758) reveals unique secreted proteins amongst haematophagous ecdysozoa. Parasit Vectors.

[57] Putnam N.H., Srivastava M., Hellsten U., Dirks B., Chapman J., Salamov A., Terry A., Shapiro H., Lindquist E., Kapitonov V.V. (2007). Sea anemone genome reveals ancestral eumetazoan gene repertoire and genomic organization. Science.

[58] Cole E.B., Miller D., Rometo D., Greenberg R.M., Brömme D., Çataltepe S., Pak S.C., Mills D.R., Silverman G.A., Luke C.J. (2004). Identification and activity of a lower eukaryotic serine proteinase inhibitor (serpin) from *Cyanea capillata*: Analysis of a jellyfish serpin, jellypin. Biochemistry.

[59] Roberts T.H., Hejgaard J., Saunders N.F.W., Cavicchioli R., Curmi P.M.G. (2004). Serpins in unicellular Eukarya, Archaea, and Bacteria: Sequence analysis and evolution. J. Mol. Evol..

[60] Zang X., Maizels R.M. (2001). Serine proteinase inhibitors from nematodes and the arms race between host and pathogen. Trends Biochem. Sci..

