# Supplementary material for: Genetic Diversity of Serine Protease Inhibitors in Myxozoan (Cnidaria, Myxozoa) Fish Parasites
Source: Microorganisms. 2020 Sep 29;8(10):1502. doi: 10.3390/microorganisms8101502 (PMC7650755; doi:10.3390/microorganisms8101502)
Supplement: Supplementary file 1 [file microorganisms-08-01502-s001.zip › Table_S2_M&M.docx]

***Supplementary Material***

Genetic diversity of serine protease inhibitors in myxozoan (Cnidaria, Myxozoa) fish parasites

Edit Eszterbauer, Dóra Sipos, Győző L. Kaján, Dóra Szegő, Ivan Fiala, Astrid S. Holzer and Pavla Bartošová-Sojková

Correspondence: Edit Eszterbauer, eszterbauer.edit@atk.hu

**Materials and Methods**

*PCR verification of* in silico *identified myxozoan serpins*

Total RNA of *M. cerebralis* and *M. pseudodispar* TAMs stored in RNAlater (Sigma-Aldrich) was extracted (including on-column DNase digestion with an RNase-Free DNase Set, Qiagen) using RNeasy Mini Kit (Qiagen) according to manufacturer’s instructions. The concentration of RNA extracts stabilized with Protector RNase Inhibitor (Merck, Roche) was estimated with Qubit 3.0 Fluorometer system (Thermo Fisher Scientific, Life Technologies).

The unknown mRNA regions of *M. cerebralis* serpin 3 (Mc-S3) and serpin 5 (Mc-S5) were identified using the cDNA synthesis system of 5′/3′ RACE Kit, 2^nd^ Generation (Merck, Roche) followed by high fidelity PCR systems (Titanium Taq PCR Kit and Advantage 2 PCR Enzyme System, Takara Bio, respectively) as per the manufacturer’s manual. PCR products either were sequenced directly or cloned using the CloneJET PCR Cloning Kit (Thermo Fisher Scientific). In the latter case, plasmids were transformed to One Shot TOP10/P3 Competent Cells (Thermo Fisher Scientific, Invitrogen). Plasmids were purified with NucleoSpin Plasmid DNA Purification Kit (Macherey-Nagel). The coding region of Mc-S3 and Mc-S5 were further examined on three independent *M. cerebralis* TAM samples using QuantiTect Reverse Transcription Kit (Qiagen), or ExiLERATE LNA cDNA Synthesis Kit (Exiqon), followed by Titanium Taq PCR Kit (Takara Bio). The total volume of the PCR was 25 µl, which contained 30 to 60 ng of template cDNA or gDNA (in 0.5 to 2 µl), 250 nM of each primer, 200 µM of dNTP (Merck, Sigma-Aldrich). The amount of other components corresponded to the manufacturers’ recommendations.

For *S. molnari* and *M. lieberkuehni*, total DNA was extracted using the phenol-chloroform protocol after the proteinase K (Serva) digestion. Total RNA was extracted using the Nucleospin RNA Kit (Macherey-Nagel) following manufacturer's instructions. cDNA synthesis was completed using the Transcriptor High Fidelity cDNA synthesis Kit (Merck, Roche) following manufacturer's protocol. Each PCR (in 25 μl total volume) consisted of 1x Taq Buffer, 250 μM of dNTP, 10 pmol of each primer, 1 U of Taq-Purple polymerase (Top-Bio), 1 μl of DNA (50 to 150 ng) and RNAase/DNAase free H_2_O. Cloning of PCR products was done as described previously [1]. In summary, PCR products were purified using the Gel/PCR DNA Fragments Extraction Kit (Geneaid Biotech Ltd) and cloned into the pDrive Vector with a PCR Cloning Kit (Qiagen) and transformed into TOP10 chemically competent *E. coli* cells (Thermo Fisher Scientific, Life Technologies). Plasmid DNA was purified using a High Pure Plasmid Isolation Kit (Merck, Roche).

For *M. cerebralis* serpins, PCR profiles were the following: denaturation at 95°C for 1 min, 35 cycles of 95°C for 30 sec, 50 to 68°C for 30 sec, 68°C for 50 sec and the final extension at 68°C for 1 min. Annealing temperature varied by oligonucleotides (Table S2); it was 60°C in general, but 55°C for primer Serp3a-F, 63°C for Serp5-F, 68°C for Serp5_318F, and 50°C for primer pairs Mc-S6_13F – McSerp_dsign_R. For *M. pseudodispar*, PCR profile was: 10 min denaturation at 95°C, 35 cycles of 50 sec at 95°C, 50 sec at 52°C, 1 min 20 sec at 72°C, and a final extension at 72°C for 5 min. For *S. molnari,* PCR profile was: DNA denaturation at 95°C for 5 min, 35 cycles of the following three steps: 50 sec at 95°C, 65°C for 50 sec (60°C for the 2^nd^ part of Sm-S2), 1 min at 68°C and a final extension at 68°C for 8 min. For *M. lieberkuehni,* AccuPower HotStart PCR PreMix (Bioneer) with 10 pmol of each oligonucleotides and 100 ng of template DNA was used for amplification following the manufacturer’s manual. PCR profile was: denaturation at 95°C for 3 min, 40 cycles of 50 sec at 95 °C, 50 sec at 55°C, 1 min 30 sec at 72°C, and a final extension at 72°C for 10 min.

Purified PCR products were Sanger sequenced with BigDye Terminator v3.1 Cycle Sequencing Kit (Thermo Fisher Scientific) using primers listed in Table S2, and detected on an Applied Biosystems Genetic Analyzer 3500 (Thermo Fisher Scientific). Consensus sequences were obtained using Geneious Prime 2019.2.3 (Biomatters Ltd.) and the Staden program package 2.0.0.b11 [2].

**Table S2.** Oligonucleotides used for PCR, RACE and/or DNA sequencing. P: PCR; R5: 5’RACE; R3: 3’RACE; S: sequencing.

| Primer name | Sequence (5’ - 3’) | Application | Serpin name (Species) |
| --- | --- | --- | --- |
| Serp1a_107F | CAGCGAGTATCTATTTTGCGC | P, S | Mc-S1 (*Myxobolus cerebralis*) |
| Serp1a_1016R | ACAACTGTAGCAGCTGAAGC | P, S |  |
| Serp1_768F | TCAACCGAAATTGTGATTTGAGC | S |  |
| Serp3a-F_276F | CGTGAATCGATTGGAGTCGT | R3 | Mc-S3 (*Myxobolus cerebralis*) |
| Serp3b-RF_1084F | GGCCCTCCTTTGCGAAATAC | R3, S |  |
| Serp3_28F | ACTTGGCATATCATGGATCTTAGC | P, S |  |
| McS3_1185cR | CATTAATATTATTGTTGAAGCTGCA | P, S |  |
| McS3_920R | AACATGTCTCGGGCACCCATTATTTG | S |  |
| Serp3_332R | AAGAAATATTCTGTCGACCCTTTCA | S |  |
| Serp4b_14F | TGATAAGTCCGCAATTGCACG | P, S | Mc-S4 (*Myxobolus cerebralis*) |
| Serp4a_1001R | AGCGGCGGCAACTACG | P, S |  |
| Serp4_845F | GGAGTGTTTGGAGAGCATGAAA | S |  |
| Serp5_318F | AGATTGTCTATTGGCTATGAATACC | R3 | Mc-S5 (*Myxobolus cerebralis)* |
| Serp5_398F | AGCCATTGGAAACAGATACG | R3 |  |
| McS5_507R | GATGTAAAACGTCTCAAGCATAGTATTCGA | R5 |  |
| Serp5-R_520R | TCGTATCTGTTTCCAATGGCT | R5 |  |
| Serp5_318FR | GGTATTCATAGCCAATAGACAATCT | R5, S |  |
| McS5_13cF | ATTTTAGTTAAGCTGAAGGTGAA | P, S |  |
| McS5_1255cR | CAAACAATTAGTAAAATCTTTGGC | P, S |  |
| Serp5-F_167F | AGAAATGGGCGCTAAAGGGA | P, R3, S | Mc-S5 & Mc-S6 (*Myxobolus cerebralis)* |
| Serp5_509R | GAGATGTAAAACGTCTCAAGCATAG | S |  |
| McS6_13F | CTGAACGTGAAGAAAAACAAAATGAAAC | P, S | Mc-S6 (*Myxobolus cerebralis)* |
| McSerp_dsign_R | CAAATATCAGDAMCAKAAAMGGHC | P, S | Mc-S1, Mc-S4, Mc-S6 (*Myxobolus cerebralis)* |
| MpSerp1_ORF153_1430R | GATCCGTTATCCCAGCAATG | P, S | Mp-S1 (*Myxobolus pseudodispar*) |
| MpSerp1_373F | TGTCAAGAAATCTGACGGCTC | P, S |  |
| MpSerp1_698F | CAATTCTAACAAGAGTCCACAAAC | S |  |
| MpSerp2_974F | TTTATTGTGAAACAGGGAGA | P, S | Mp-S2 (*Myxobolus pseudodispar*) |
| MpSerp2_ORF56_1444R | CAATTATTCCAGCTACAAAGAGTGG | P, S |  |
| SmolSerp1F2 | AAAGATGGTCTTATCGCACA | P, S | Sm-S1 (*Sphaerospora molnari*) |
| SmolSerp1R2 | TTAGCGATCGAAGTTACTCC | P, S |  |
| SmolSerp 2xb_F | TTCGAGTGAATGTCGTTCTAATTTTGA | P, S | Sm-S2 (*Sphaerospora molnari*) |
| SmolSerp2xb_R | TTTGGAAG ACAGTCTTGAATATCC | P, S |  |
| SmolSerp 2_2F | TCAAGACTGTGCTTCCAAAT | P, S |  |
| SmolSerp2_2R | TCATGGTCACAACAAACAGA | P, S |  |
| Mlieb_Serpin F2 | TCCTCAATGGATTTGGGTGTT | P, S | Ml-S1 (*Myxidium lieberkuehni*) |
| Mlieb_Serpin R2 | GCGGCTACAACTCCCTCTTC | P, S |  |

**References**

1. Bartošová-Sojková, P.; Lövy, A.; Reed, C.C.; Lisnerová, M.; Tomková, T.; Holzer, A.S.; Fiala, I. Life in a rock pool: Radiation and population genetics of myxozoan parasites in hosts inhabiting restricted spaces. *PLoS One* **2018**, *13*, e0194042, doi:10.1371/journal.pone.0194042.

2. Staden, R.; Beal, K.F.; Bonfield, J.K. The Staden package, 1998. *Methods Mol Biol* **2000**, *132*, 115–130.
