# Supplementary figures and images for: Genetic Diversity of Serine Protease Inhibitors in Myxozoan (Cnidaria, Myxozoa) Fish Parasites
Source: Microorganisms. 2020 Sep 29;8(10):1502. doi: 10.3390/microorganisms8101502 (PMC7650755; doi:10.3390/microorganisms8101502)

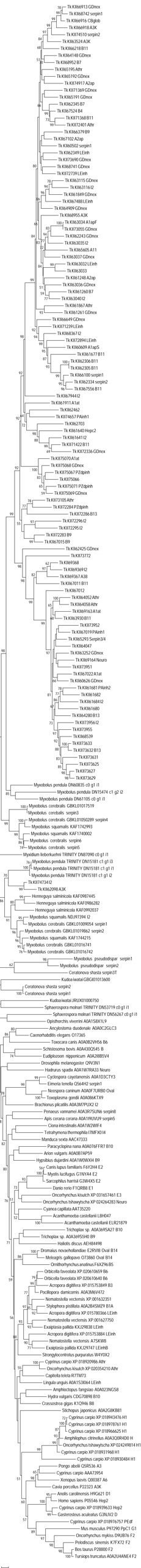

Supplement: Supplementary file 1 [file microorganisms-08-01502-s001.zip › Figure_S1.PDF]
